# 2,2′-(4-Methyl-4*H*-1,2,4-triazole-3,5-di­yl)dibenzene­sulfonamide

**DOI:** 10.1107/S1600536812006113

**Published:** 2012-02-17

**Authors:** Tasleem Akhtar, Waseeq Ahmad Siddiqui, Adnan Ashraf, M. Nawaz Tahir

**Affiliations:** a University of Sargodha, Department of Chemistry, Sargodha, Pakistan; b University of Sargodha, Department of Physics, Sargodha, Pakistan

## Abstract

In the title compound, C_15_H_15_N_5_O_4_S_2_, the dihedral angles between the central 1,2,4-triazole ring and the pendant benzene rings are 55.61 (10) and 68.59 (10)°; the dihedral angle between the benzene rings is 63.66 (9)°. Intra­molecular N—H⋯N and N—H⋯O hydrogen bonds generate *S*(7) and *S*(12) rings, respectively. In the crystal, sheets extending in the (101) plane arise, with the mol­ecules linked by C—H⋯O, N—H⋯N and N—H⋯O inter­actions. A C—H⋯π inter­action further consolidates the structure.

## Related literature

For background to benzisothia­zole derivatives, see: Siddiqui *et al.* (2007 ▶); Siddiqui, Ahmad, Khan *et al.* (2008 ▶); Siddiqui, Ahmad, Siddiqui & Parvez (2008 ▶). For related crystal structures, see: Carlsen *et al.* (1995 ▶). For graph-set notation, see: Bernstein *et al.* (1995 ▶).
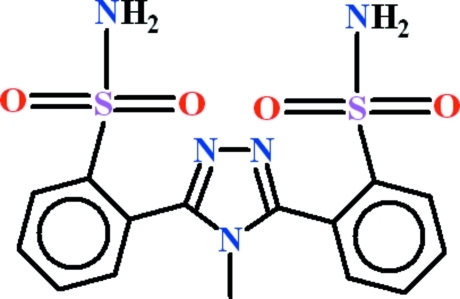


## Experimental

### 

#### Crystal data

C_15_H_15_N_5_O_4_S_2_
*M* *_r_* = 393.44
Monoclinic, 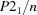

*a* = 13.4190 (6) Å
*b* = 6.9043 (2) Å
*c* = 19.0498 (9) Å
β = 102.243 (2)°
*V* = 1724.80 (12) Å^3^
*Z* = 4
Mo *K*α radiation
μ = 0.34 mm^−1^
*T* = 296 K
0.35 × 0.25 × 0.22 mm

#### Data collection

Bruker Kappa APEXII CCD diffractometer
Absorption correction: multi-scan (*SADABS*; Bruker, 2005 ▶) *T*_min_ = 0.915, *T*_max_ = 0.938
15158 measured reflections
4055 independent reflections
2526 reflections with *I* > 2σ(*I*)
*R*_int_ = 0.060

#### Refinement

*R*[*F* ^2^ > 2σ(*F* ^2^)] = 0.059
*wR*(*F* ^2^) = 0.158
*S* = 1.03
4055 reflections
239 parameters
H atoms treated by a mixture of independent and constrained refinement
Δρ_max_ = 0.63 e Å^−3^
Δρ_min_ = −0.66 e Å^−3^

### 

Data collection: *APEX2* (Bruker, 2009 ▶); cell refinement: *SAINT* (Bruker, 2009 ▶); data reduction: *SAINT*; program(s) used to solve structure: *SHELXS97* (Sheldrick, 2008 ▶); program(s) used to refine structure: *SHELXL97* (Sheldrick, 2008 ▶); molecular graphics: *ORTEP-3 for Windows* (Farrugia, 1997 ▶) and *PLATON* (Spek, 2009 ▶); software used to prepare material for publication: *WinGX* (Farrugia, 1999 ▶) and *PLATON*.

## Supplementary Material

Crystal structure: contains datablock(s) global, I. DOI: 10.1107/S1600536812006113/hb6629sup1.cif

Structure factors: contains datablock(s) I. DOI: 10.1107/S1600536812006113/hb6629Isup2.hkl

Supplementary material file. DOI: 10.1107/S1600536812006113/hb6629Isup3.cml

Additional supplementary materials:  crystallographic information; 3D view; checkCIF report

## Figures and Tables

**Table 1:** Hydrogen-bond geometry (Å, °) *Cg*1 and *Cg*3 are the centroids of the C7/N2/C8/N3/N4 and C10–C15 rings, respectively.

*D*—H⋯*A*	*D*—H	H⋯*A*	*D*⋯*A*	*D*—H⋯*A*
N1—H1*A*⋯O3	0.82 (4)	2.33 (4)	3.082 (4)	153 (3)
N1—H1*B*⋯N4^i^	0.95 (4)	1.96 (4)	2.899 (4)	171 (3)
N5—H5*A*⋯O4^ii^	0.94 (4)	2.10 (4)	3.011 (4)	164 (3)
N5—H5*B*⋯N3	0.83 (4)	2.14 (4)	2.876 (4)	148 (4)
C9—H9*B*⋯O2^iii^	0.96	2.17	2.990 (3)	142
C14—H14⋯*Cg*3^iv^	0.93	2.68	3.583 (4)	163

Symmetry codes: (i) 
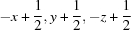
; (ii) 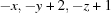; (iii) 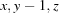; (iv) 
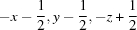
.

**Fig. 1.**
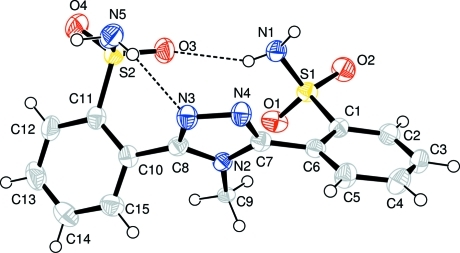
View of the title compound with displacement ellipsoids drawn at the 50% probability level. The dotted lines represent the intramolecular hydrogen bonds.

**Fig. 2.**
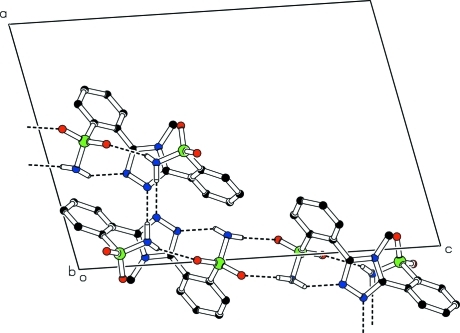
The partial packing (PLATON; Spek, 2009) which shows that molecules form two dimensional polymeric network in the plane (101).

## supplementary crystallographic information

### Comment

In continuation to our research work on the synthesis of benzisothiazole derivatives (Siddiqui, Ahmad, Khan *et al.*, 2008; Siddiqui, Ahmad, Siddiqui & Parvez, 2008), the title compound (I), (Fig. 1) is prepared from hydrazine and commercial source of saccharin.

The crystal structures of 4-methyl-3,5-diphenyl-4*H*-1,2,4-triazolethe has been published which is also related to (I).

In (I), the phenyl rings A (C1–C6), B (C10—C15) and the 4-methyl-4*H*- 1,2,4-triazole moiety C (C7–C9/N2–N4) are planar with r. m. s. deviation of 0.0079 Å, 0.0051 Å and 0.0310 Å, respectively. The dihedral angle between A/B, A/C and B/C is 63.66 (9)°, 68.59 (1)° and 55.61 (10)°, respectively. There exist intramolecular H-bonding of N—H···N and N—H···O types (Table 1, Fig. 1) forming S (7) and S (12) ring motifs (Bernstein *et al.*, 1995), respectively. There exist intermolecular H-bondings of C—H···.O, N—H···N and N—H···O types (Table 1, Fig. 2) which consolidates the molecules in the form two-dimensional polymeric network extending along the (101) plane. There exist C—H···π (Table 1) interactions which also play role in establishing the structure.

### Experimental

For the synthesis of title compound, hydrazine monohydrate and saccharin were used as the starting materials following a reported procedure (Siddiqui *et al.*, 2007). Colourless needles of (I) suitable for X-ray crystallographic study were grown from methanol at room temperature. m. p. = 483–484 K. FT—IR: (KBr, cm^-1^): 3296, 3263 (NH and NH_2_), 2987 (Ar. CH), 1651 (C═ N), 1541 (NH def.), 1454 (CH def.), 1315, 1151 (SO_2_).

### Refinement

The coordinates of H-atoms of amino groups were refined. The H-atoms were positioned geometrically (C—H = 0.93–0.96 Å) and refined as riding with *U*_iso_(H) = *xU*_eq_(C, N), where *x* = 1.5 for methyl groups and *x* = 1.2 for all other H-atoms.

### Crystal data

**Table:**

C_15_H_15_N_5_O_4_S_2_	*F*(000) = 816
*M**_r_* = 393.44	*D*_x_ = 1.515 Mg m^−^^3^
Monoclinic, *P*2_1_/*n*	Mo *K*α radiation, λ = 0.71073 Å
Hall symbol: -P 2yn	Cell parameters from 2526 reflections
*a* = 13.4190 (6) Å	θ = 2.1–27.9°
*b* = 6.9043 (2) Å	µ = 0.34 mm^−^^1^
*c* = 19.0498 (9) Å	*T* = 296 K
β = 102.243 (2)°	Prism, colourless
*V* = 1724.80 (12) Å^3^	0.35 × 0.25 × 0.22 mm
*Z* = 4	

### Data collection

**Table:**

Bruker Kappa APEXII CCD diffractometer	4055 independent reflections
Radiation source: fine-focus sealed tube	2526 reflections with *I* > 2σ(*I*)
Graphite monochromator	*R*_int_ = 0.060
Detector resolution: 7.60 pixels mm^-1^	θ_max_ = 27.9°, θ_min_ = 2.1°
ω scans	*h* = −17→17
Absorption correction: multi-scan (*SADABS*; Bruker, 2005)	*k* = −5→9
*T*_min_ = 0.915, *T*_max_ = 0.938	*l* = −24→25
15158 measured reflections	

### Refinement

**Table:**

Refinement on *F*^2^	Primary atom site location: structure-invariant direct methods
Least-squares matrix: full	Secondary atom site location: difference Fourier map
*R*[*F*^2^ > 2σ(*F*^2^)] = 0.059	Hydrogen site location: inferred from neighbouring sites
*wR*(*F*^2^) = 0.158	H atoms treated by a mixture of independent and constrained refinement
*S* = 1.03	*w* = 1/[σ^2^(*F*_o_^2^) + (0.0762*P*)^2^] where *P* = (*F*_o_^2^ + 2*F*_c_^2^)/3
4055 reflections	(Δ/σ)_max_ < 0.001
239 parameters	Δρ_max_ = 0.63 e Å^−^^3^
0 restraints	Δρ_min_ = −0.66 e Å^−^^3^

### Special details

**Table:**

Geometry. Bond distances, angles *etc*. have been calculated using the rounded fractional coordinates. All su's are estimated from the variances of the (full) variance-covariance matrix. The cell e.s.d.'s are taken into account in the estimation of distances, angles and torsion angles
Refinement. Refinement of *F*^2^ against ALL reflections. The weighted *R*-factor *wR* and goodness of fit *S* are based on *F*^2^, conventional *R*-factors *R* are based on *F*, with *F* set to zero for negative *F*^2^. The threshold expression of *F*^2^ > σ(*F*^2^) is used only for calculating *R*-factors(gt) *etc*. and is not relevant to the choice of reflections for refinement. *R*-factors based on *F*^2^ are statistically about twice as large as those based on *F*, and *R*- factors based on ALL data will be even larger.

### Fractional atomic coordinates and isotropic or equivalent isotropic displacement parameters (Å^2^)

**Table:**

	*x*	*y*	*z*	*U*_iso_*/*U*_eq_	
S1	0.05268 (6)	1.38253 (11)	0.12266 (4)	0.0315 (3)	
S2	−0.01231 (6)	1.09870 (12)	0.38488 (4)	0.0358 (3)	
O1	−0.04824 (15)	1.3040 (3)	0.11187 (13)	0.0438 (8)	
O2	0.06566 (19)	1.5630 (3)	0.08924 (13)	0.0470 (9)	
O3	0.00582 (18)	1.2207 (3)	0.32800 (12)	0.0444 (8)	
O4	−0.06624 (19)	1.1753 (4)	0.43601 (13)	0.0509 (9)	
N1	0.0933 (2)	1.4069 (4)	0.20696 (16)	0.0362 (9)	
N2	0.03107 (18)	0.9092 (3)	0.18915 (13)	0.0266 (8)	
N3	0.12821 (19)	0.8730 (4)	0.29572 (14)	0.0340 (8)	
N4	0.18700 (19)	0.9443 (4)	0.25015 (14)	0.0327 (8)	
N5	0.0966 (2)	1.0255 (5)	0.42979 (16)	0.0423 (10)	
C1	0.1333 (2)	1.2155 (4)	0.08967 (16)	0.0286 (9)	
C2	0.1717 (2)	1.2741 (5)	0.03096 (17)	0.0369 (11)	
C3	0.2396 (3)	1.1553 (5)	0.00495 (18)	0.0408 (11)	
C4	0.2696 (3)	0.9836 (5)	0.03807 (19)	0.0447 (12)	
C5	0.2307 (2)	0.9214 (5)	0.09593 (18)	0.0379 (11)	
C6	0.1620 (2)	1.0357 (4)	0.12269 (16)	0.0292 (9)	
C7	0.1277 (2)	0.9661 (4)	0.18635 (16)	0.0287 (9)	
C8	0.0343 (2)	0.8550 (4)	0.25834 (16)	0.0286 (9)	
C9	−0.05428 (11)	0.8905 (4)	0.13235 (8)	0.0214 (8)	
C10	−0.05358 (11)	0.7942 (3)	0.28739 (8)	0.0307 (10)	
C11	−0.08105 (11)	0.8909 (3)	0.34553 (8)	0.0330 (10)	
C12	−0.16186 (11)	0.8275 (3)	0.37385 (8)	0.0438 (11)	
C13	−0.2172 (3)	0.6684 (6)	0.3451 (2)	0.0513 (14)	
C14	−0.1922 (3)	0.5723 (5)	0.2878 (2)	0.0480 (14)	
C15	−0.1123 (3)	0.6349 (5)	0.25875 (19)	0.0400 (11)	
H1A	0.063 (3)	1.330 (5)	0.2279 (18)	0.0435*	
H1B	0.164 (3)	1.434 (5)	0.2203 (18)	0.0435*	
H2	0.15207	1.39242	0.00900	0.0442*	
H3	0.26444	1.19303	−0.03496	0.0490*	
H4	0.31685	0.90708	0.02156	0.0538*	
H5	0.25064	0.80228	0.11706	0.0454*	
H5A	0.085 (3)	0.941 (5)	0.466 (2)	0.0509*	
H5B	0.128 (3)	0.971 (6)	0.402 (2)	0.0509*	
H9A	−0.11300	0.85753	0.15121	0.0321*	
H9B	−0.04191	0.79024	0.10032	0.0321*	
H9C	−0.06624	1.01082	0.10672	0.0321*	
H12	−0.17881	0.89259	0.41243	0.0524*	
H13	−0.27155	0.62553	0.36427	0.0613*	
H14	−0.22961	0.46418	0.26857	0.0577*	
H15	−0.09730	0.57006	0.21947	0.0480*	

### Atomic displacement parameters (Å^2^)

**Table:**

	*U*^11^	*U*^22^	*U*^33^	*U*^12^	*U*^13^	*U*^23^
S1	0.0307 (4)	0.0274 (4)	0.0364 (5)	0.0039 (3)	0.0074 (3)	−0.0018 (3)
S2	0.0423 (5)	0.0348 (5)	0.0332 (5)	−0.0007 (3)	0.0148 (4)	−0.0013 (3)
O1	0.0263 (11)	0.0412 (13)	0.0614 (16)	0.0034 (10)	0.0036 (11)	−0.0089 (12)
O2	0.0642 (16)	0.0277 (12)	0.0519 (16)	0.0059 (11)	0.0186 (13)	0.0070 (11)
O3	0.0603 (15)	0.0357 (13)	0.0417 (14)	0.0005 (11)	0.0209 (12)	0.0067 (11)
O4	0.0629 (16)	0.0508 (15)	0.0467 (15)	0.0013 (12)	0.0293 (12)	−0.0079 (12)
N1	0.0323 (15)	0.0407 (17)	0.0381 (17)	−0.0015 (12)	0.0128 (12)	−0.0058 (13)
N2	0.0264 (12)	0.0258 (13)	0.0283 (14)	0.0021 (10)	0.0076 (10)	−0.0016 (11)
N3	0.0288 (13)	0.0396 (16)	0.0343 (15)	0.0012 (12)	0.0084 (11)	0.0036 (12)
N4	0.0281 (13)	0.0381 (15)	0.0329 (15)	0.0027 (11)	0.0089 (11)	0.0039 (12)
N5	0.0444 (17)	0.050 (2)	0.0328 (17)	−0.0027 (15)	0.0087 (13)	−0.0014 (14)
C1	0.0262 (15)	0.0314 (17)	0.0273 (16)	0.0010 (13)	0.0038 (12)	−0.0009 (13)
C2	0.0411 (18)	0.0367 (19)	0.0317 (18)	−0.0037 (15)	0.0053 (14)	0.0017 (15)
C3	0.0415 (19)	0.051 (2)	0.0340 (19)	−0.0078 (16)	0.0171 (15)	−0.0034 (17)
C4	0.043 (2)	0.049 (2)	0.047 (2)	0.0085 (17)	0.0205 (16)	−0.0061 (18)
C5	0.0394 (18)	0.0351 (19)	0.043 (2)	0.0096 (14)	0.0170 (15)	0.0021 (15)
C6	0.0262 (15)	0.0291 (17)	0.0326 (17)	−0.0019 (13)	0.0069 (13)	−0.0053 (14)
C7	0.0262 (15)	0.0273 (16)	0.0332 (17)	0.0045 (12)	0.0080 (13)	−0.0027 (13)
C8	0.0303 (15)	0.0260 (16)	0.0302 (17)	0.0036 (12)	0.0077 (13)	−0.0024 (13)
C9	0.0180 (13)	0.0237 (15)	0.0206 (14)	−0.0009 (11)	−0.0002 (11)	−0.0030 (12)
C10	0.0279 (15)	0.0306 (17)	0.0346 (18)	0.0041 (13)	0.0092 (13)	0.0040 (14)
C11	0.0305 (16)	0.0385 (19)	0.0300 (17)	0.0013 (13)	0.0065 (13)	0.0054 (14)
C12	0.0427 (19)	0.057 (2)	0.0361 (19)	−0.0083 (17)	0.0182 (15)	0.0004 (17)
C13	0.040 (2)	0.062 (3)	0.055 (2)	−0.0150 (18)	0.0174 (18)	0.010 (2)
C14	0.040 (2)	0.043 (2)	0.060 (3)	−0.0113 (16)	0.0085 (18)	0.0022 (19)
C15	0.0381 (18)	0.0349 (19)	0.048 (2)	−0.0011 (15)	0.0111 (16)	−0.0046 (16)

### Geometric parameters (Å, º)

**Table:**

S1—O1	1.433 (2)	C4—C5	1.384 (5)
S1—O2	1.427 (2)	C5—C6	1.390 (4)
S1—N1	1.592 (3)	C6—C7	1.466 (4)
S1—C1	1.784 (3)	C8—C10	1.466 (3)
S2—O3	1.433 (2)	C10—C11	1.408 (2)
S2—O4	1.432 (3)	C10—C15	1.395 (4)
S2—N5	1.610 (3)	C11—C12	1.381 (2)
S2—C11	1.783 (2)	C12—C13	1.373 (4)
N2—C7	1.367 (4)	C13—C14	1.378 (5)
N2—C8	1.362 (4)	C14—C15	1.376 (6)
N2—C9	1.405 (3)	C2—H2	0.9300
N3—N4	1.382 (4)	C3—H3	0.9300
N3—C8	1.315 (4)	C4—H4	0.9300
N4—C7	1.313 (4)	C5—H5	0.9300
N1—H1B	0.95 (4)	C9—H9A	0.9600
N1—H1A	0.82 (4)	C9—H9B	0.9600
N5—H5B	0.83 (4)	C9—H9C	0.9600
N5—H5A	0.94 (4)	C12—H12	0.9300
C1—C6	1.408 (4)	C13—H13	0.9300
C1—C2	1.387 (4)	C14—H14	0.9300
C2—C3	1.393 (5)	C15—H15	0.9300
C3—C4	1.363 (5)		
			
O1—S1—O2	117.89 (15)	N2—C8—N3	109.2 (2)
O1—S1—N1	107.21 (15)	N3—C8—C10	125.3 (3)
O1—S1—C1	109.28 (13)	N2—C8—C10	125.5 (2)
O2—S1—N1	108.05 (15)	C11—C10—C15	117.5 (2)
O2—S1—C1	105.47 (14)	C8—C10—C15	120.7 (2)
N1—S1—C1	108.68 (14)	C8—C10—C11	121.80 (19)
O3—S2—O4	119.23 (15)	C10—C11—C12	121.03 (17)
O3—S2—N5	107.80 (15)	S2—C11—C10	120.94 (13)
O3—S2—C11	108.10 (11)	S2—C11—C12	118.04 (13)
O4—S2—N5	106.69 (15)	C11—C12—C13	120.1 (2)
O4—S2—C11	106.99 (13)	C12—C13—C14	119.9 (3)
N5—S2—C11	107.53 (14)	C13—C14—C15	120.6 (3)
C7—N2—C8	106.3 (2)	C10—C15—C14	120.9 (3)
C7—N2—C9	128.4 (2)	C1—C2—H2	120.00
C8—N2—C9	125.0 (2)	C3—C2—H2	120.00
N4—N3—C8	107.6 (2)	C2—C3—H3	120.00
N3—N4—C7	107.9 (2)	C4—C3—H3	120.00
H1A—N1—H1B	125 (3)	C3—C4—H4	120.00
S1—N1—H1A	109 (2)	C5—C4—H4	119.00
S1—N1—H1B	114 (2)	C4—C5—H5	120.00
H5A—N5—H5B	112 (4)	C6—C5—H5	120.00
S2—N5—H5B	109 (3)	N2—C9—H9A	109.00
S2—N5—H5A	108 (3)	N2—C9—H9B	109.00
C2—C1—C6	120.3 (3)	N2—C9—H9C	109.00
S1—C1—C2	116.9 (2)	H9A—C9—H9B	109.00
S1—C1—C6	122.8 (2)	H9A—C9—H9C	109.00
C1—C2—C3	119.9 (3)	H9B—C9—H9C	109.00
C2—C3—C4	119.9 (3)	C11—C12—H12	120.00
C3—C4—C5	121.0 (3)	C13—C12—H12	120.00
C4—C5—C6	120.5 (3)	C12—C13—H13	120.00
C5—C6—C7	117.8 (3)	C14—C13—H13	120.00
C1—C6—C5	118.5 (3)	C13—C14—H14	120.00
C1—C6—C7	123.7 (3)	C15—C14—H14	120.00
N2—C7—N4	109.0 (3)	C10—C15—H15	120.00
N4—C7—C6	124.6 (3)	C14—C15—H15	119.00
N2—C7—C6	126.4 (3)		
			
O1—S1—C1—C2	114.3 (2)	S1—C1—C6—C7	1.0 (4)
O1—S1—C1—C6	−68.8 (3)	C2—C1—C6—C5	1.2 (4)
O2—S1—C1—C2	−13.3 (3)	C2—C1—C6—C7	177.8 (3)
O2—S1—C1—C6	163.6 (2)	C1—C2—C3—C4	−1.2 (5)
N1—S1—C1—C2	−129.0 (2)	C2—C3—C4—C5	2.4 (6)
N1—S1—C1—C6	47.9 (3)	C3—C4—C5—C6	−1.7 (5)
O3—S2—C11—C10	43.59 (19)	C4—C5—C6—C1	−0.1 (5)
O3—S2—C11—C12	−136.53 (16)	C4—C5—C6—C7	−176.9 (3)
O4—S2—C11—C10	173.16 (17)	C1—C6—C7—N2	69.9 (4)
O4—S2—C11—C12	−6.95 (19)	C1—C6—C7—N4	−111.8 (3)
N5—S2—C11—C10	−72.55 (19)	C5—C6—C7—N2	−113.4 (3)
N5—S2—C11—C12	107.34 (18)	C5—C6—C7—N4	64.8 (4)
C8—N2—C7—N4	1.1 (3)	N2—C8—C10—C11	−121.9 (3)
C8—N2—C7—C6	179.6 (3)	N2—C8—C10—C15	59.2 (4)
C9—N2—C7—N4	−173.4 (3)	N3—C8—C10—C11	55.4 (4)
C9—N2—C7—C6	5.1 (4)	N3—C8—C10—C15	−123.6 (3)
C7—N2—C8—N3	−1.4 (3)	C8—C10—C11—S2	2.2 (3)
C7—N2—C8—C10	176.2 (2)	C8—C10—C11—C12	−177.68 (19)
C9—N2—C8—N3	173.3 (2)	C15—C10—C11—S2	−178.9 (2)
C9—N2—C8—C10	−9.2 (4)	C15—C10—C11—C12	1.3 (3)
C8—N3—N4—C7	−0.5 (3)	C8—C10—C15—C14	177.2 (3)
N4—N3—C8—N2	1.2 (3)	C11—C10—C15—C14	−1.7 (4)
N4—N3—C8—C10	−176.4 (2)	S2—C11—C12—C13	179.8 (2)
N3—N4—C7—N2	−0.4 (3)	C10—C11—C12—C13	−0.3 (3)
N3—N4—C7—C6	−178.9 (3)	C11—C12—C13—C14	−0.2 (5)
S1—C1—C2—C3	176.5 (3)	C12—C13—C14—C15	−0.3 (6)
C6—C1—C2—C3	−0.6 (5)	C13—C14—C15—C10	1.3 (6)
S1—C1—C6—C5	−175.6 (2)		

### Hydrogen-bond geometry (Å, º)

Cg1 and Cg3 are the centroids of the C7/N2/C8/N3/N4 and C10–C15 rings, respectively.

**Table:**

*D*—H···*A*	*D*—H	H···*A*	*D*···*A*	*D*—H···*A*
N1—H1*A*···O3	0.82 (4)	2.33 (4)	3.082 (4)	153 (3)
N1—H1*B*···N4^i^	0.95 (4)	1.96 (4)	2.899 (4)	171 (3)
N5—H5*A*···O4^ii^	0.94 (4)	2.10 (4)	3.011 (4)	164 (3)
N5—H5*B*···N3	0.83 (4)	2.14 (4)	2.876 (4)	148 (4)
C9—H9*B*···O2^iii^	0.96	2.17	2.990 (3)	142
C14—H14···*Cg*3^iv^	0.93	2.68	3.583 (4)	163

Symmetry codes: (i) −*x*+1/2, *y*+1/2, −*z*+1/2; (ii) −*x*, −*y*+2, −*z*+1; (iii) *x*, *y*−1, *z*; (iv) −*x*−1/2, *y*−1/2, −*z*+1/2.
